# *Aspergillus bertholletius sp. nov.* from Brazil Nuts

**DOI:** 10.1371/journal.pone.0042480

**Published:** 2012-08-27

**Authors:** Marta H. Taniwaki, John I. Pitt, Beatriz T. Iamanaka, Daniele Sartori, Marina V. Copetti, Arun Balajee, Maria Helena P. Fungaro, Jens C. Frisvad

**Affiliations:** 1 Centro de Ciência e Qualidade de Alimentos, Instituto de Tecnologia de Alimentos, Campinas, São Paulo, Brazil; 2 CSIRO Animal, Food and Health Sciences, Sydney, New South Wales, Australia; 3 Centro de Ciências Biológicas, Universidade Estadual de Londrina, Londrina, Paraná, Brazil; 4 Departamento de Tecnologia e Ciência de Alimentos, Universidade Federal de Santa Maria, Santa Maria, Rio Grande do Sul, Brazil; 5 Mycotic Diseases Branch, Centers for Disease Control and Prevention, Atlanta, Georgia, United States of America; 6 Department of Systems Biology, Technical University of Denmark, Lyngby, Denmark; Catalan Institute for Water Research (ICRA), Spain

## Abstract

During a study on the mycobiota of brazil nuts (*Bertholletia excelsa*) in Brazil, a new *Aspergillus* species, *A. bertholletius,* was found, and is described here. A polyphasic approach was applied using morphological characters, extrolite data as well as partial β-tubulin, calmodulin and ITS sequences to characterize this taxon. *A. bertholletius* is represented by nineteen isolates from samples of brazil nuts at various stages of production and soil close to *Bertholletia excelsa* trees. The following extrolites were produced by this species: aflavinin, cyclopiazonic acid, kojic acid, tenuazonic acid and ustilaginoidin C. Phylogenetic analysis using partial β-tubulin and camodulin gene sequences showed that *A. bertholletius* represents a new phylogenetic clade in *Aspergillus* section *Flavi.* The type strain of *A. bertholletius* is CCT 7615 ( = ITAL 270/06 = IBT 29228).

## Introduction

Brazil nuts are one of the most important products extracted from the Amazon rainforest region. Trees of *Bertholletia excelsa* grow wild, reaching up to 60 meters, take 12 years to bear fruit and may live up to 500 years. The trees are found in groves of 50–100 individuals and the groves are separated by up to 1 km. Pollination is by wild, large bodied bees, especially *Euglossinae* species [1]. The Amazon rainforest has multiple ecosystems with a huge biodiversity. It has an important role in the global weather balance and is the location of many native peoples. The equatorial climate is hot and humid, with an average temperature of 26°C and relative humidity of 80–95%.

Several studies on the mycobiota of brazil nuts have been carried out. The most commonly isolated species are *Aspergillus flavus, A. nomius, A. parasiticus, A. niger, A. tamarii, A. pulverulentus, A. flavo-furcatis, Penicilliumglabrum, P. citrinum, Rhizopus* spp. and *Fusarium oxysporum* [2], [3], [4], [5].

Among these species, a major concern relates to those within *Aspergillus* section *Flavi*, because some of them have the potential for aflatoxin production. A major challenge in brazil nut production is controlling the high rate of contamination by species within *Aspergillus* section *Flavi* and hence the potential for high aflatoxin production.

The taxonomy of this section is still highly complex and continually evolving. Phylogenetic analysis based on sequence data from β-tubulin and calmodulin genes revealed that *Aspergillus* section *Flavi* includes seven main clades (*A. flavus* clade, *A. tamarii* clade, *A. nomius* clade, *A. alliaceus* clade, *A. togoensis* clade, *A. leporis* clade, and *A. avenaceus* clade), with 20 or more taxa [6]. According to these authors, the main clades are well defined. However, many subclades are represented by a single isolate and further collections and studies are needed to clarify speciation in this section.

During a course of studies on the mycobiota of brazil nuts, a new *Aspergillus* taxon in *Aspergillus* section *Flavi* was found in soil and several brazil nut samples collected at various stages of the production chain. This species is described here as *Aspergillus bertholletius sp. nov.* It does not produce aflatoxin.

## Materials and Methods

### Fungal isolation from brazil nuts samples and soil

A total of 290 brazil nut samples (174 nuts and 116 shells) each of approximately 2 kg were collected in the Amazon region and São Paulo State, Brazil. Besides that, 28 samples of soil (each approximately 200 g) were collected from Amazon rainforest close to *Bertholletia excelsa* trees. The sampling was carried out together with the Brazilian Ministry of Agriculture and all necessary permits were obtained for the described field studies. The taxonomic study is supported by Brazilian Resolution of Genetic Heritage Management Council (MMA/CGEN 21/06).

Approximately 100 g of shelled nuts and 100 g of shells were disinfected separately by immersion in 0.4% sodium hypochlorite solution for 1 min. Fifty pieces of nuts or shells were plated onto Dichloran 18% Glycerol agar (DG18), according to the methodology of Pitt and Hocking [7]. Plates were incubated for 5 days at 25°C.

For soil samples, under aseptic conditions, samples (25 g) were weighed and sterile peptone water (0.1%; 225 ml) was added. Aliquots were serially diluted and spread plated onto Dichloran 18% Glycerol agar. The plates were incubated at 25°C for 7 days, according to Pitt and Hocking [7]. All isolateswith the appearance of belonging to *Aspergillus* section *Flavi* were isolated onto Czapek yeast extract agar [7]and incubated at 25°C for 7 days.

### Morphological examination

The fungi were examined on standard identification media for *Aspergillus* species, namely Czapek yeast extract agar (CYA), malt extract agar (MEA), Aspergillus flavus and parasiticus agar (AFPA) and 25% glycerol nitrate agar (G25N) [7] at 25°C and also at 37°C and 42°C on CYA. The incubation time for all media and conditions was 7 days.

The standard conditions used for the description of *Aspergillus bertholletius* are taken from Pitt and Hocking [7]. Capitalised colours are from the Methuen Handbook of Colour [8].

### Molecular analysis

Isolates were cultivated in yeast extract and lactose (YEL) solid medium for seven days. From each culture, a suspension of approximately 10^7^ conidia suspended in Tween 80 (2.5 ml) was inoculated into bottles containing YEL liquid (50 ml), and incubated in a shaker (180 rpm) at 28°C for 16 to 24 h. After incubation, mycelia were collected by vacuum filtration and washed in sterile water. Nucleic acids were extracted according to Azevedo *et al.* [9], and treated with ribonucleaseA (20 µg/ml). Partial amplification of the ß-tubulin gene was performed using standard amplification reactions and the following primer pair: Bt2a (5′ GGT AAC CAA ATC GGT GCT TTC 3′) and Bt2b (5′ ACC CTC AGT GTA GTG ACC CTT GGC 3′), as described by Glass and Donaldson [10]. Part of the calmodulin gene region was amplified using the cmd5 (5′ CCG AGT ACA AGG AGG CCT TC 3′) and cmd6 (5′ CCG ATA GAG GTC ATA ACG TGG 3′) primers previously reported by Hong et al. [11]. Similarly, the ITS1–5.8S–ITS2 region of rDNA was amplified with the primer ITS1 (5′ TCCGTAGGTGAACCTGCGG3′) and ITS4 (TCCTCCGCTTATTGATATGC3′) [12]. Fragments generated by PCRwere purified with Wizard® SV Gel and PCR Clean-Up System (Promega). The amplicons were submitted to direct sequencing in both directions (forward and reverse) with a Big Dye Terminator Cycle Sequencing Standart kit Version 3.1 (Applied Biosystems, Foster City, Calif., USA) under the following conditions: denaturation at 95°C for 60 s, followed by 30 cycles of denaturation at 95°C for 20 s, annealing at 50°C for 15 s, extension at 60°C for 1.5 min,and a final extension at 60°C for 3 min. A volume of HiDiformamide(10 µL) was added to the sequencing products, which were processed in an ABI 3500XL Genetic Analyser (Applied Biosystems, Foster City, Calif., USA. The sequences obtained were aligned to those type species sequences from *Aspergillus* section *Flav*i deposited in the NCBI database (http://www.ncbi.nlm.nih.gov/)using Clustal W [13]. The software package MEGA5 [14] was used to construct a neighbour joining tree [15].

### Extrolite analysis

The cultures were analysed by HPLC with diode arraydetection according to the method of Frisvad and Thrane [16] as modified by Houbraken *et al.* [17]. The isolates were analysed on CYA and YES agar using three agar plugs. Five plugs of each agar medium were taken and pooled into same vial for extraction with 0.75 ml of a mixture of ethyl acetate/dichloromethane/methanol (3∶2∶1) (v/v/v) with 1% (v/v) formic acid.

### Nomenclature

1. The electronic version of this article in Portable Document Format (PDF) in a work with an ISSN or ISBN will represent a published work according to the International Code of Nomenclature for algae, fungi, and plants, and hence the new names contained in the electronic publication of a PLOS ONE article are effectively published under that Code from the electronic edition alone, so there is no longer any need to provide printed copies.

In addition, new names contained in this work have been submitted to MycoBank from where they will be made available to the Global Names Index. The unique MycoBank number can be resolved and the associated information viewed through any standard web browser by appending the MycoBank number contained in this publication to the prefix http://www.mycobank.org/MB. The online version of this work is archived and available from the following digital repositories: PubMed Central, LOCKSS.

2. Repository of ***Aspergillus bertholletius*** Taniwaki, Pitt & Frisvad 2012 sp. nov. [urn:lsid:mycobank.org: 800125]

## Results and Discussion

Of 290 brazil nut samples (nuts and shells), 15 samples showed the presence of *Aspergillus bertholletius*, with an incidence ranging from 2 to 46% infection after direct plating on DG18. In total, 65 isolates were found from shells and nuts and from soil close to *Bertholletia excelsa* trees. The origin of the *Aspergillus bertholletius* isolates is shown in Table 1, and the incidence of *A. bertholletius* in the samples throughout the brazil nut chain in Table 2. Most samples were not infected by *A. bertholletius*. However, one sample from a street market in the Amazon region was highly infected, with 46% and 36% of nuts and shells infected. Of 28 samples of soil from areas adjacent to *B. excelsa* trees, only one was contaminated with *A. bertholletius,* showing a count of 8.0×10^3^ CFU/g. Isolation of *A. bertholletius* from brazil nuts and soil may have been underestimated because colonies of *A. bertholletius* on DG18 are similar to those of *A. tamarii*. Distinctions were found after incubating isolates on CYA at 37°C, where colonies of *A. bertholletius* are 5 to 15 mm in diameter, while those of *A. tamariii* are 50 mm or more in diameter [7]. When cultured on AFPA, *A. bertholletius* is readily recognised from *A. tamarii* on AFPA by a cream colony reverse, unlike the dark brown characteristic of *A. tamarii.* On the other hand, *A. bertholletius* differs from *A. flavus* and *A. parasiticus* which give an orange reverse colour on AFPA due to the production of aspergilic acid or noraspergillic acid which react with ferric ammonium citrate present in the medium [7].

**Table 1:** *Aspergillus bertholletius* isolates from brazil nuts (nuts and shell) and soil from Amazonian rainforest.

Code	Substrate	Local of collect (States)
116	Nut	Market (Amazon)
118	Nut	Market (Amazon)
259	Shell	Market (Amazon)
262	Shell	Market (Amazon)
270/06	Soil	Rainforest close to *Bertholletia excelsa* tree (Amazon)
271/06	Soil	Rainforest close to *Bertholletia excelsa* tree (Amazon)
272/06	Soil	Rainforest close to *Bertholletia excelsa* tree (Amazon)
273/06	Soil	Rainforest close to *Bertholletia excelsa* tree (Amazon)
274/06	Soil	Rainforest close to *Bertholletia excelsa* tree (Amazon)
275/06	Soil	Rainforest close to *Bertholletia excelsa* tree (Amazon)
276/06	Soil	Rainforest close to *Bertholletia excelsa* tree (Amazon)
277/06	Soil	Rainforest close to *Bertholletia excelsa* tree (Amazon)
396	Shell	Supermarket (São Paulo)
412	Shell	Supermarket (São Paulo)
1784	Shell	Processing (Pará)
1875	Shell	Processing (Pará)
4891	Shell	Rainforest (Pará)
7106	Nut	Processing (Amazon)
7153	Nut	Market (Amazon)
7155	Nut	Market (Amazon)
7156	Nut	Market (Amazon)
7157	Nut	Market (Amazon)
7161	Nut	Market (Amazon)
7162	Nut	Market (Amazon)
7163	Nut	Market (Amazon)
7164	Nut	Market (Amazon)
7178	Nut	Market (Amazon)
7179	Nut	Market (Amazon)
7180	Nut	Market (Amazon)
7181	Nut	Market (Amazon)
7183	Nut	Market (Amazon)
7184	Nut	Market (Amazon)
7187	Nut	Market (Amazon)
7189	Nut	Market (Amazon)
7190	Nut	Market (Amazon)
7191	Nut	Market (Amazon)
7192	Nut	Market (Amazon)
7193	Nut	Market (Amazon)
7194	Nut	Market (Amazon)
7195	Nut	Market (Amazon)
7196	Nut	Market (Amazon)
7197	Nut	Market (Amazon)
7202	Shell	Market (Amazon)
7203	Shell	Market (Amazon)
7204	Shell	Market (Amazon)
7207	Shell	Market (Amazon)
7212	Shell	Market (Amazon)
7213	Shell	Market (Amazon)
7215	Shell	Market (Amazon)
7218	Shell	Market (Amazon)
7219	Shell	Market (Amazon)
7224	Shell	Market (Amazon)
7227	Shell	Market (Amazon)
7232	Shell	Market (Amazon)
7233	Shell	Market (Amazon)
7234	Shell	Market (Amazon)
7236	Shell	Market (Amazon)
7242	Shell	Market (Amazon)
7244	Shell	Market (Amazon)
7245	Shell	Market (Amazon)
7370	Nut	Market (Amazon)
7428	Nut	Market (Amazon)
7651	Nut	Market (Pará)
7687	Nut	Market (Pará)
7707	Nut	Market (Pará)

**Table 2:** Incidence of *A. bertholletius* in brazil nut samples.

Origin	Nuts		Shell	
	N° of samples/n° of positive samples	Range of infection (%)	N° of samples/n° of positive samples	Range of infection (%)
Rainforest	59/0	0	59/1	2
Processing	40/2	2	21/3	2–4
Street market (Amazonian region)	54/6	2–46	32/2	4–36
Supermarket (São Paulo)	21/0	0	4/1	4

**Figure 1:**
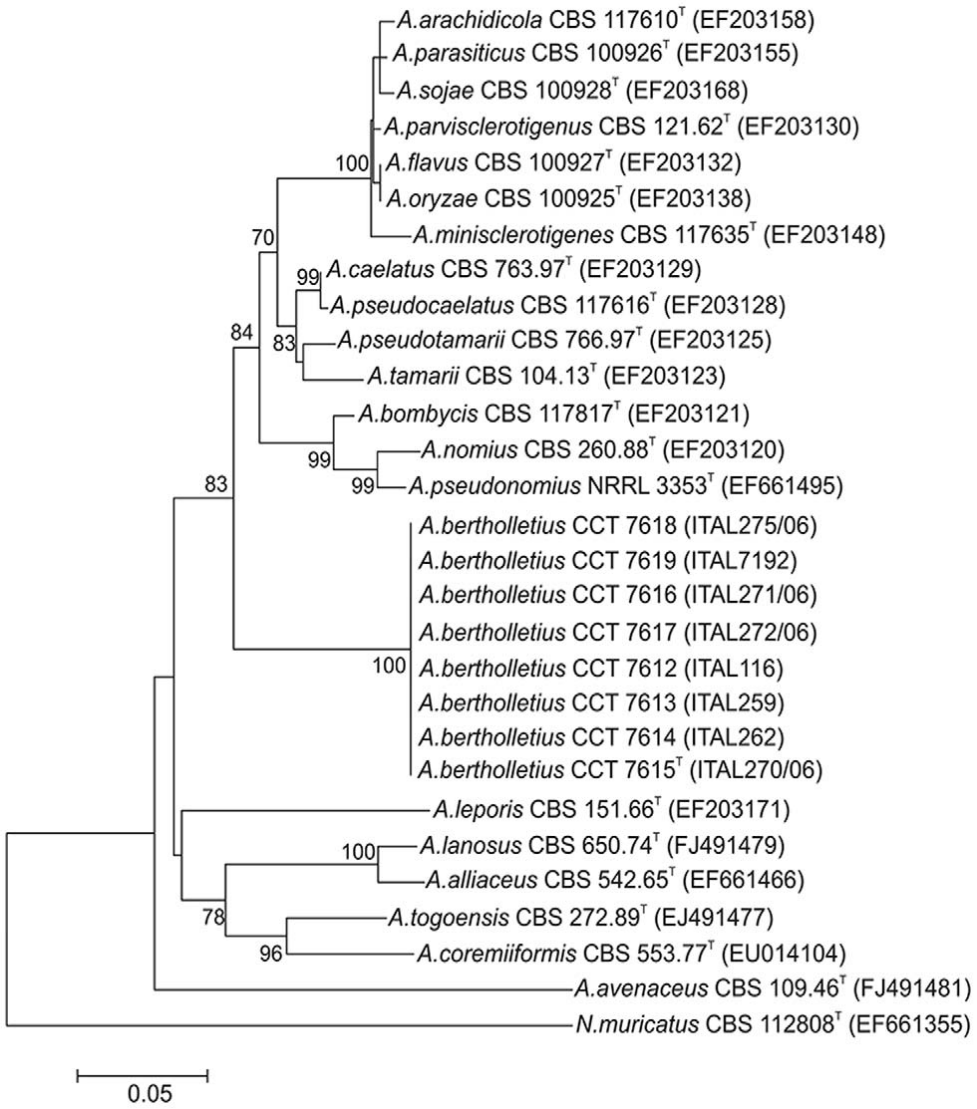
Neighbour joining tree reconstructed from the β-tubulin gene sequences aligned with corresponding sequences of *Aspergillus* section *Flavi* type species deposited in public databases. Numbers at branch nodes refer to bootstrap values (1000 replicates), only values of >70% are shown. The nucleotide sequence from the type strain of *A. bertholletius* CCT 7615 ( = ITAL 270/06) has been deposited in the GenBank databases under GenBank accession no. JQ744022.

**Figure 2:**
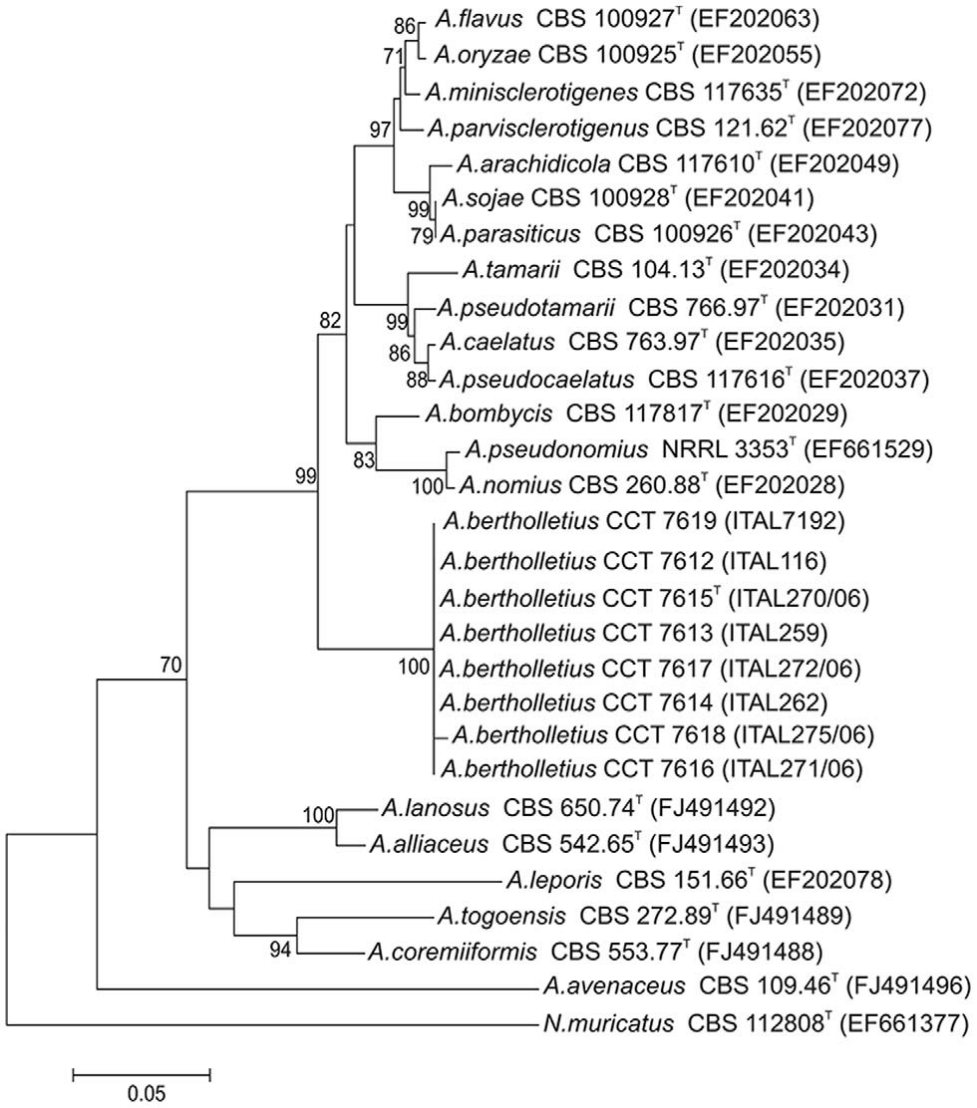
Neighbour joining tree reconstructed from the calmodulin partial gene sequences aligned with corresponding sequences of *Aspergillus* section *Flavi* type species deposited in public databases. Numbers at branch nodes refer to bootstrap values (1000 replicates), only values of >70% are shown. The calmodulin nucleotide sequences from the type strain of *A. bertholletius* CCT 7615 ( = ITAL 270/06) has been deposited in the GenBank databases under accession no. JX 198674.

**Figure 3:**
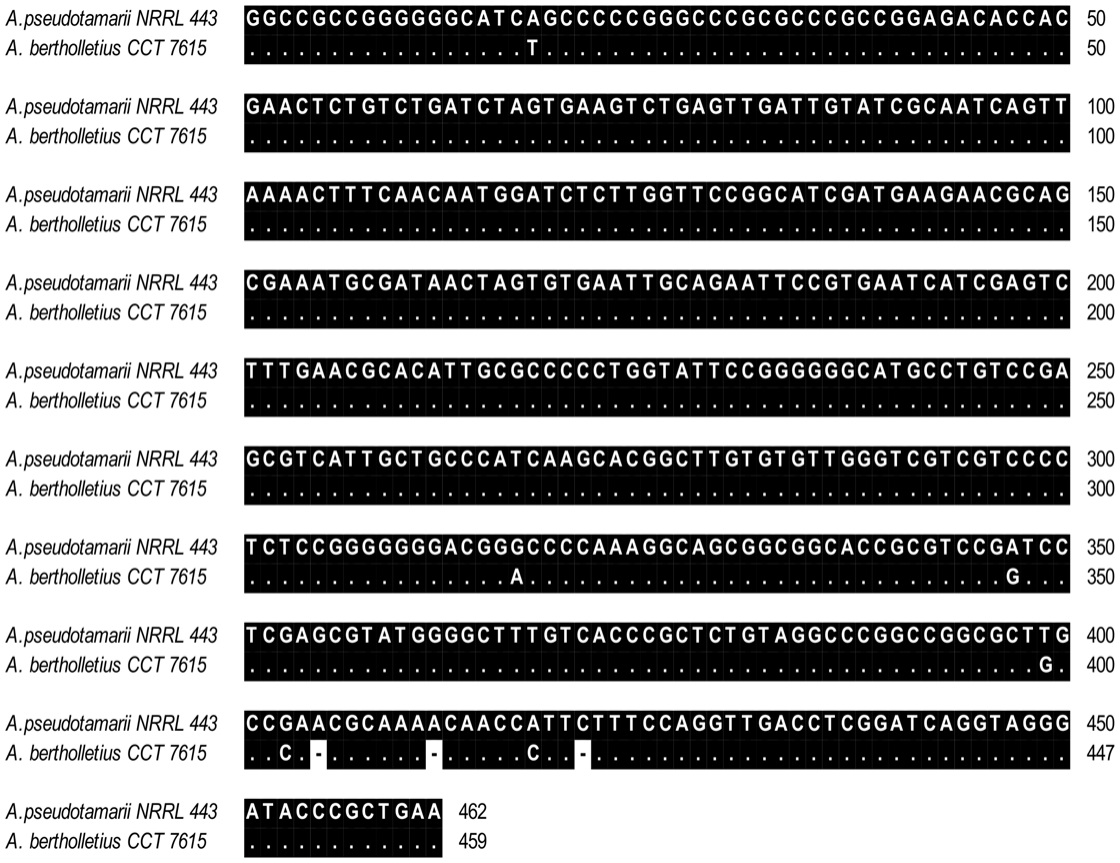
Nucleotide sequence alignment of a 459-bp fragment of the ITS-5.8S-ITS region of *Aspergillus bertholletius* (accession no. JX 198673, present study) and *A. pseudotamarii* (AF004931).

Apart from very slow growth at 37°C, the morphology of strains of *A. bertholletius* are consistent with placement within *Aspergillus* section *Flavi.* However, that striking difference in growth rate at 37°C correlates well with the distinct separation of *A. bertholletius* from other species in section *Flavi*in a neighbour joining tree derived from b-tubulin and calmodulin sequences. The nucleotide sequence data of β-tubulin and calmodulin genes matched in showing that the *A. bertholletius* isolates represent a new phylogenetic clade in *Aspergillus* section *Flavi* (Figure 1 and 2). In addition, *A. bertholletius* was also differentiated from all other known *Aspergillus* when analyzing the ITS1–5.8S–ITS2 region. A comparison of a 459-bp fragment from this region of *A. bertholletius* relative to *A. pseudotamarii*, the taxon with the most similar sequence indicated by BLASTn tool, revealed 6 nucleotide substitutions and 3 insertion/deletions (Figure 3).

Metabolite analysis indicated that *A. bertholletius* does not produce aflatoxins. However, one strain, the ex type strain, CCT 7615, produced O-methylsterigmatocystin, indicating that *A. bertholletius* may have silent genes for aflatoxin production. All strains produced the mycotoxin cyclopiazonic acid or its precursors and five of 18 strains examined produced the mycotoxin tenuazonic acid. Other metabolites produced were kojic acid (17/18 strains), ustilaginoidin C (9/18 strains) and indole alkaloids (16/18 strains). The isolates exhibited a unique profile of metabolites, consistent with production by an undescribed species.

*Aspergillus bertolletius* shares the production of cyclopiazonic acid with *A. flavus, A. minisclerotigenes, A. oryzae, A. parvisclerotigenus, A. pseudocaelatus, A. pseudotamarii*, and *A. tamarii*. It shares the ability to produce tenuazonic acid with *A. caelatus* and *A. nomius* and O-methylsterigmatocystin with all aflatoxin producers. It shares kojic acid with all species in *Aspergillus* section *Flavi*, except *A. avenaceus* [6].

**Figure 4:**
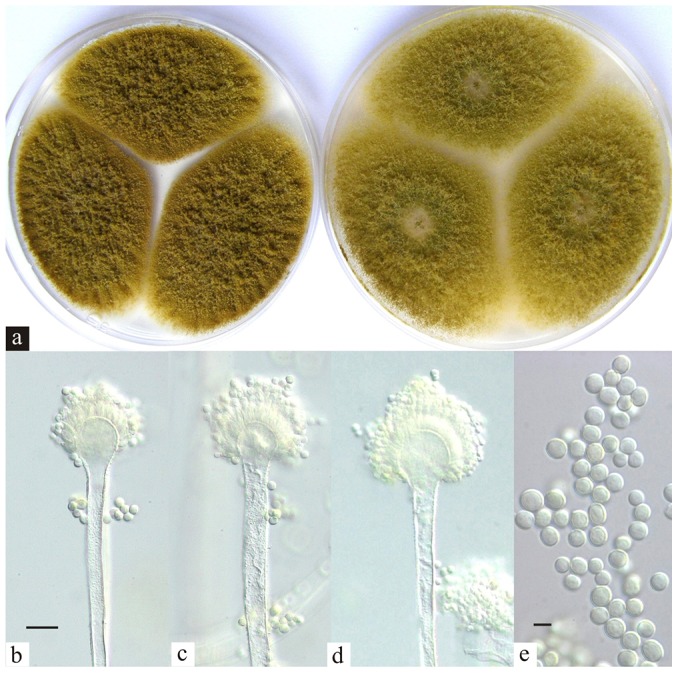
*Aspergillus bertholletius.* (a) Colonies on Czapek yeast extract agar and malt extract agar after 7 days incubation at 25°C; (b, c, d) conidial heads, bar = 10 µm; (d) conidia, bar = 5 µm.

Figure 4 shows the morphology of A. bertholletius colonies on Czapek yeast extract agar and malt extract agar after 7 days incubation at 25°C and the conidial heads.

### *Aspergillus bertholletius* Taniwaki, Pitt & Frisvad *sp. nov*. [urn:lsid:mycobank.org: 800125]

#### Etymology

Named from the generic epithet of the brazil nut tree *Bertholletia excelsa*, the known habitat for this species.

#### Holotype

CCT 7615 in Coleção de Cultura Tropical (Campinas, Brazil) is designated as the holotype of *Aspergillus bertholletius*. It was isolated from soil close to *Bertholletia excelsa* trees, Instituto de Tecnologia de Alimentos, Campinas, Brazil, 2006. Cultures derived from type include ITAL 270/06 (where ITAL is the culture collection of Instituto de Tecnologia de Alimentos, Campinas, Brazil), and IBT 29228 (where IBT is the culture collection of the Technical University of Denmark, Lyngby, Denmark).

#### Diagnosis

This species differs from of species in *Aspergillus* section *Flavi* by slow growth on CYA at 37°C, linoleum brown conidia *en masse*, a unique profile of secondary metabolites and a distinct DNA sequence in the region of the ß-tubulin and calmodulin genes.

#### Description

Colonies on CYA 60–70 mm in diameter, often almost covering the Petri dish, deep but velutinous; margins entire, narrow; mycelium inconspicuous; conidiogenesis heavy, brown near Linoleum Brown (M. 5E7); exudate and soluble pigment absent; reverse uncoloured to pale brown. Colonies on MEA 60–70 mm in diameter, similar to on CYA, but conidia slightly more green than on CYA, olive brown near Khaki (M. 4D5-E7); reverse pale.

Colonies on G25N 25 mm in diameter, low, often heavily sporing in colours near those on MEA; reverse pale.

Colonies on CYA at 37°C 5–15 mm in diameter, sometimes with brown sporulation.

Conidiophores borne from surface or aerial hyphae, 70–150×6–8 µm, with very thin, smooth walls; vesicles spherical, 10–17(–20) µm in diameter, bearing uncrowded phialides; phialides ampulliform, large and broad, 10–14×5–6(–7) µm; conidia uniform in size and shape, spherical, 5.5–6.5 µm in diameter, with finally spinose walls, borne in long, tangled chains. Sclerotia are not produced on any media.

#### Other isolates examined

ITAL 116 ( = CCT 7612), ITAL 259 ( = CCT 7613),ITAL 262( = CCT 7614 = IBT 31739),ITAL 271/06 ( = CCT 7616 = IBT 30618), ITAL 272/06 ( = CCT 7617 = IBT 30617), ITAL 273/06 ( = IBT 30619), ITAL 275/06 ( = CCT 7618 = IBT 29227), ITAL 7157 ( = IBT 31548), ITAL 7179 ( = IBT 31554), ITAL 7180 ( = IBT 31555), ITAL 7189 ( = IBT 31151), ITAL 7191 ( = IBT 31553), ITAL 7192 ( = CCT 7619), ITAL 7193( = IBT 31549), ITAL 7194 ( = IBT 31556), ITAL 7195 ( = IBT 31557), ITAL 7196 ( = IBT 31546) and ITAL 7197( = IBT 31500), all from nuts of *Bertholletia excelsa,* the brazil nut tree and soil close to the tree.

## Conclusion

*A. bertholletius* represents a new important phylogenetic clade in *Aspergillus* section *Flavi* applying a polyphasic approach using morphological characters, extrolite data, β-tubulin and calmodulin partial gene sequences.
